# Evidence of Human-designated Antiretroviral (ARV) Drug Residues in Broiler Chicken, Domestic Pigs, and Animal Feeds in Tanzania

**DOI:** 10.21203/rs.3.rs-5107085/v1

**Published:** 2024-09-19

**Authors:** Zuhura Kimera, Peter Shimo, Emmanuel Ballandya, Mecky Isaac Matee, Lisa Adams

**Affiliations:** Muhimbili University of Health and Allied Sciences; Tanzania Government Chemistry Laboratory; Muhimbili University of Health and Allied Sciences; Muhimbili University of Health and Allied Sciences; Center for Global Health Equity, The Geisel School of Medicine at Dartmouth, Hanover, NH 03755, USA

**Keywords:** Antiretroviral (ARV), Drug Residues, Broiler Chicken, Domestic Pigs, Animal Feeds, Tanzania

## Abstract

**Background:**

Recent reports have indicated the use of antiretroviral (ARV) drugs to boost animal production in neighboring Uganda, with further reports of use in several African countries.

**Methods:**

This cross-sectional study was conducted in nine districts in Tanzania, and involved screening for the presence of three first-line ARVs (lamivudine, nevirapine, and efavirenz) residues in the muscle and blood of domestic pigs and broiler chickens, and in sampled animal feed and water. Residues were determined using liquid chromatography and mass spectrometry (LC-MS/MS). The method involved calibration of the lower limit of quantitation (LLOQ) and limit of detection (LOD). ARVs were detected and quantified using the Multiple Reaction Monitoring (MRM) system.

**Results:**

131 (66.8%) of the 196 samples of muscle, blood, and animal feed were found to contain lamivudine residues, with the highest concentration detected in domestic pig blood and muscle (7.58mg/kg) and the lowest concentration (0.01 mg/kg) in broiler chicken feed. There was a significant relationship between the presence of lamivudine by sample type and sample origin (p=0.000). Nevirapine and efavirenz drugs were not detected in any of the collected samples. No ARV residues were detected in water samples (n=37).

**Conclusion:**

This study confirms the use of ARVs in animal production in Tanzania as evidenced by the presence of residues in animal feeds. We found lamivudine residues in domestic pigs and broiler chickens at concentrations higher than those recently reported in other East African studies. Farmers living with HIV may be using ARVs from their prescribed medications, which may lead to poor adherence and the emergence of drug resistance. Besides direct human and animal health issues, these residues in animal feeds and animal excreta can lead to environmental contamination leading to several negative impacts. We recommend a total ban on human-designated ARVs in animal production and advocate for comprehensive studies and monitoring systems across African countries to reveal potential societal and other reasons for their use and provide comprehensive solutions using One Health approaches.

## Background

Human immunodeficiency virus (HIV) infection is one of the major global health problems, particularly in sub-Saharan African (SSA) countries (World Helath Organization, 2023). In 2022, approximately 39 million people were living with HIV globally of whom 25.6 million, over two-thirds of all HIV infections, were living in SSA (UNAIDS, 2023). According to UNAIDS 2024, statistics Tanzania has a total of 1,700, 000 adults and children living with human immunodeficiency virus (HIV), of whom 1, 400,000 are on antiretroviral (ARV) drugs (UNAIDS, 2024). The coverage of pregnant women who receive ARV for prevention of mother-to-child transmission (PMTCT) of HIV infection is 98%, averting 18,000 new HIV infections annually(?) (UNAIDS, 2024).

According to the national guidelines for the management of HIV/AIDS (United Republic of Tanzania, 2019), the first-line ARV regimen for treating and preventing HIV infection recommended for adults and adolescents consists of tenofovir disoproxil fumarate (TDF) + lamivudine (3TC) (or emtricitabine, FTC) + efavirenz (EFV). For the past 20 years, Tanzania has received significant support for purchasing antiretroviral drugs (ARVs) from international organizations and initiatives, witnessing a remarkable increase in the number of people living with HIV (PLWH) on ARVs from 22% in 2009 to over 98% in 2023 (Tarimo, 2023). Thanks to the support of international agencies and organizations, ARVs are widely and freely available at all levels of service delivery from dispensaries to health centers and public and private hospitals (Mziray et al. 2021).

The fact that ARVs are widely available and free, coupled with weak regulations, has led to their uncontrolled use beyond their intended goal. In East African countries, particularly in Uganda, ARVs have been reported to be used for growth promotion in pigs and poultry, but also for the treatment of viral infections in animals such as African swine fever, fermentation of alcohol, and cooking beans (Nakato et al., 2020; Ndoboli et al., 2021; Obonyo, 2013; Odongo, 2016; Opoka, 2018; Valentine, 2013). Recent studies from Uganda have detected a considerable amount of ARV residues in food animals, and the source of the drugs was PLWH who self-treat their animals (Nakato et al., 2020; Ndoboli et al., 2021) and black-market sales for profit by unscrupulous traders (Kasang et al., 2011). There are also reports of misuse of ARVs in neighboring Kenya (Valentine et al 2013) and several other communities in East Africa (Hatari 2013; Oketch 2016; URN 2017).

Despite the widespread usage and ease of access to ARV drugs in Tanzania and the allegations of ARV misuse in animal farming, there is no data to ascertain the occurrence of active pharmaceutical ingredients in food animals, animal feeds, and water sources. This study aims to determine the presence of lamivudine (3TC), efavirenz (EFV), and nevirapine (NVP) residues in broiler poultry and domestic pigs, animal feeds, and water sources. This study aimed to provide information on whether ARV residues are found in animal products and feeds and the surrounding environment We hypothesize that the use of ARVs in animal feeds for growth promotion will lead to their deposition in animal tissues, and when excreted through urine or feces will contaminate the surrounding environment and potentially remain bioactive, leading to wide-ranging chemical and biological effects with potential impact on public health and the environment.

## Methods

### Study area

**Figure 1:**
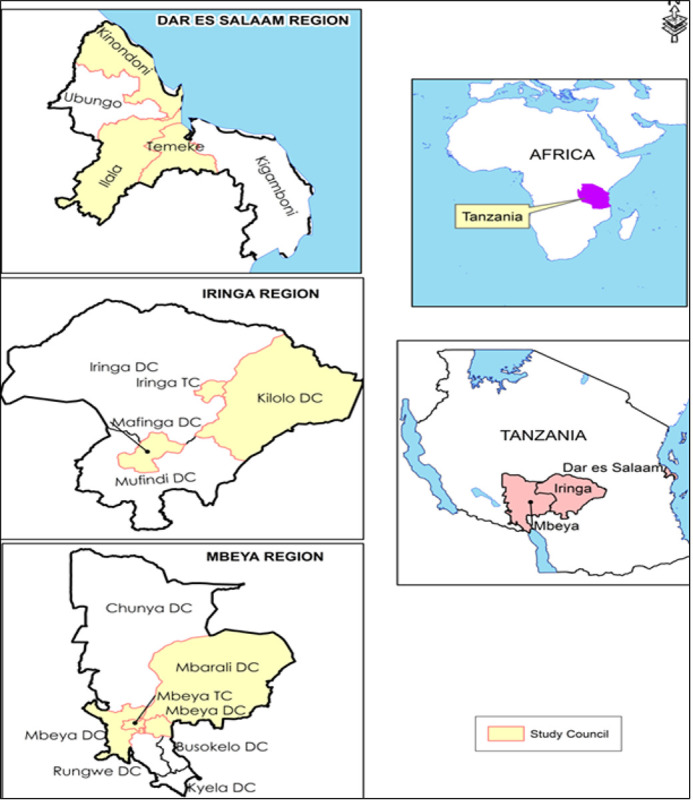
Map showing the Dar es Salaam, Mbeya, and Iringa regions with the nine districts involved in this study

This cross-section study was conducted in nine districts in Dar es Salaam, Mbeya, and Iringa regions as shown in the map (Fig. 1). The Dar es Salaam region is the largest city in Tanzania and the biggest consumer of chickens and domestic pigs originating from all parts of the country (Mubito et al., 2014). Mbeya, Iringa, Morogoro, Rukwa, and Ruvuma regions from the Southern Highlands are the leading domestic pig producers contributing to 54% of the domestic pigs in Tanzania (Kimbi et al., 2015).

### Sampling strategy

The study involved nine districts; Temeke, Ilala, and Kinondoni in the Dar es Salaam region, Mbeya Town Council, Mbeya District Council and Mbalizi in the Mbeya region, and Iringa Municipal Council, Kilolo and Mafinga in Iringa region. In each of the selected districts, the abattoir/slaughter slabs were identified for sampling because they receive animals from different localities. Broiler chicken samples were collected from the Dar es Salaam region only because the city is a major producer and market for broiler poultry. Information on the origin of the animal was collected before taking blood and tissue to avoid having samples originating from a single source.

### Sample collection

About 10 mL of blood was collected using BD Vacutainer EDTA tubes and 25 g of muscle tissue was collected from the freshly slaughtered animals. The samples were labeled and immediately transported in an icebox at 4°C to the Department of Microbiology and Immunology at the Muhimbili University of Health and Allied Sciences for storage at −20°C pending analysis.

### Laboratory procedures

Determination of ARVs in the collected samples was conducted at the Tanzania Government Chemistry Laboratory Authority (GCLA) Head Office in Dar es Salaam, using Liquid Chromatography with tandem mass spectrometry (LC-MS-MS), which is a powerful analytical technique that combines the separating power of liquid chromatography with the highly sensitive and selective mass analysis capability of triple quadrupole mass spectrometry.

### Chemicals

Reagents included the internal standards (IS) of efavirenz, nevirapine, and lamivudine purchased from Millipore Sigma Aldrich (USA). These panels of ARVs were selected because they are the first-line drugs for the management of HIV/AIDS in Tanzania (United Republic of Tanzania, 2019). These ARVs are available in mono-component type and fixed-dose combination (FDC). Acetonitrile, methanol, formic acid, water, and all LC-MS hyper grade for the mobile phase, were purchased from Bio solve (Dieuze, France). The HPLC-grade acetonitrile and methanol that were used for protein precipitation, and hydrochloric acid used as analytical reagent were purchased from Merck Research laboratory (Whitehouse Station, NJ, USA).

### Conditions of the Chromatographic and Mass-Spectrometric

The chromatographic and mass-spectrometric condition was set as described by (Djerada et al., 2013) with some modifications. Briefly, a standard liquid chromatographic (LC-MS) system (Q-Exactive Orbitrap Ms, UK), equipped with a cooled auto-sampler, column oven, and Xevo TQ mass spectrometer (Waters Corp., Milford, MA, USA) was used. The mobile phase (A) consisted of water + formic acid 0.1% (V/V) and the mobile phase (B) had acetonitrile + formic acid 0.1% (V/V). The mobile phase gradient was set at 0.6 mL/min flow rate; 0.0 min at 95% of mobile phase A, followed by an increase and decrease of mobile phase B of 90% from 0.0 to 5.0 min, to 5% from 5.0 to 5.1 min respectively. At mobile phase A, the flow rate was maintained at 95% of mobile phase A from 5.1 to 5.5 min. The electrospray probe was kept free from non-retained components using a 0.1-min solvent/diverts delay to waste. At the end of the spectrometric acquisition, the column eluent was again diverted to waste.

### Preparation of stock solutions, calibration standards, and quality control samples

Stock solutions of internal standard nevirapine, efavirenz, and lamivudine were prepared in HPLC-grade methanol, containing 0.005 N HCl, to obtain a final concentration of 1 mg/mL. Working solutions were diluted with a suitable volume of methanol and finally in the blank sample (< 1/9 V/V) to prepare calibration standards (STD). Quality control (QC) samples were prepared with stock solutions different from those used to prepare the calibration standards and were prepared in blank samples then kept at −20°C for subsequent use for a period not exceeding one month.

### Sample preparation

ARV drug residues in the collected samples were extracted as described by (Djerada et al., 2013). The amount of 2g or 2mL of the sample was weighed and was followed by the addition of 2 ml of 8:2 methanol: water and vortexed for 1 min. Thereafter, 4mL of acetonitrile were added and the mixture was centrifuged at 5,000 rpm for 10 min. then 1mL of the supernatant was filtered and placed in a glass vial for chromatographic separation. ARV residues in broiler chickens and pig meat and blood, animal feeds, and wastewater were qualitatively quantified by LC-MS/MS. The ARVs residues were separated on a VF-5ms (Ultra inert) 100mm × 2.1mm × 2.2 μM column and detected by Q-TOF triple quadrupole mass spectrometry (MS/MS) operating with electron energy at 35 eV. The injection volume was 5 μL. All ARV residues were detected and quantified in the Multiple Reaction Monitoring (MRM).

### Method validation

Method validation included calibration of the lower limit of quantitation (LLOQ) and limit of detection (LOD). The LLOQ was considered the lowest calibration standard detected and the LOD was the estimated concentration that gives chromatographic peaks with a ratio of 5 as described by (Djerada et al., 2013). The lower limit of quantitation (LLOQ) was estimated by decreasing the amounts of analytes in samples and was calculated as the concentration estimated from 10 ppm to 0.01 ppm. Calibration standards (n = 6) were prepared and analyzed with a minimum of six (6) independent runs for each compound at a concentration of 0.5–10 ppm. Thereafter, a six (6) point calibration curve was constructed using least squares linear or non-linear regression quadratic regression equation, with r^2^ between 0.995 and 0996. The recovery tests were performed by spiking three (3) levels 0.5, 2.5, and 5.0 ppm with recovery ranging from 76.60–91.78 for lamivudine, 71.68–116 for nevirapine and 86.88–105.72 for efavirenz. The detailed information on validated data is shown in Table 1.

### Matrix effect

The matrix effect was assessed by international standards(?) (European Medicines Agency, 2012). The absolute matrix effect was assessed for all analytes by comparing the chromatographic peak areas of spiked blank sample extracts (i.e. after protein precipitation with methanol and acetonitrile) from six different sources to peak areas obtained from the same concentration of analytes in the same composition of the extract (100 μL of methanol + 200 μL of acetonitrile + 450 ^L of water containing 0.1% formic acid) without sample. Additionally, matrix effects of the entire chromatographic run were evaluated using a post-column infusion of the analytes to ensure that no interfering peaks of the blank sample (n = 7) extract were found at the retention time corresponding to each analyte. The blank sample was extracted and injected into the LC-MS/MS system with concurrent post-column infusion of analytes.

### Recovery

Recovery was determined by comparing the peak area obtained from spiked samples with the peak area from a standard solution of all analytes in a solution of (100 μL of methanol + 200 μL of acetonitrile + 450 μL of water containing 0.1% formic acid) at the same concentrations.

## Results

A total of 196 samples were collected from nine districts within three regions. The number of samples per region was: Dar es Salaam 124, Mbeya 32, and Iringa 40. Out of 196 samples, 66.8% (n = 131) were found to have lamivudine residues while no ARV nevirapine or efavirenz residues were detected. No ARV residues were detected from all of the water samples (n = 37). The occurrence of ARV lamivudine per region is presented in Table 2.

### Presence of ARV lamivudine residues based on district of origin

By district, Temeke had the highest number of positive samples (90%, n = 18) followed by Kinondoni (83%, n = 58), compared to other districts across the three regions (Table 3). Both Temeke and Kinondoni districts are in the Dar es Salaam region.

### Distribution of occurrence of ARV lamivudine residues based on type of sample

As shown in Table 4 below, all samples of broiler chicken meat (100%, n = 30) had lamivudine residues. The same table shows that more than half of the samples collected from broiler chicken blood, domestic pig meat, and blood and animal feeds were found to have different concentrations of lamivudine residues.

### Concentration of lamivudine residues

As shown in Table 5, higher levels of lamivudine (7.58mg/kg) were detected both in pig meat and blood, while the lowest concentration (0.12mg/kg) was detected in domestic pigs.

### Association between ARV lamivudine residues by sample type and sample origin

The level of lamivudine residues detected was significantly related to regions (p = 0.014), district of sample collection (p = 0.000) and sample type (p = 0.000) associated with origin (p = 0.009), regions (p = 0.133), or district of sample origin (p = 0.583).

## Discussion

This is the first study to report the presence of ARVs in domestic animals and commercial animal feeds in Tanzania. In Sub-Saharan Africa (SSA), there are only two such studies, and both are from Uganda (Nakato et al., 2020; Ndoboli et al., 2021). The majority of studies in SSA have focused on the use of antibiotics and other agents in animals (Founou et al., 2016; Kimera, Mshana, et al., 2020; Mshana et al., 2013).

In this study, two-thirds (66.6%, n = 131) of the 196 samples of muscle, blood, and animal feed were found to contain lamivudine residues, with the highest concentration detected in domestic pig blood and muscle (7.58mg/kg) and the lowest concentration (0.01 mg/kg) in broiler chicken feed. There was a significant relationship between the presence of lamivudine by sample type and sample origin (p = 0.000). Temeke and Kinondoni districts in Dar es Salaam had the highest prevalence of residues at 90% and 83%, respectively. We did not detect nevirapine or efavirenz in any of the tested samples.

The proportion of samples with lamivudine residues detected in this study (66.6%) is significantly higher than that reported in Uganda where efavirenz and nevirapine residues were detected in in 13.6% and 13.9% of sampled pork meat (Nakato et al., 2020). Another study conducted in Uganda detected 5.5% of saquinavir residues and 2.5% of lopinavir residues in 5.5% of pork and 2.5% of chicken samples respectively (Ndoboli et al., 2021).

There are notable differences between our study and those conducted in Uganda. The study conducted in Uganda by Nakato and colleagues found efavirenz in one-quarter of samples, while we did not find any. The likely reason we did not detect efavirenz (EFV) or nevirapine (NVP) is that our samples were collected after these drugs were excluded by WHO from the recommended first-line regimen which now consists of tenofovir, lamivudine, and dolutegravir (DTG) (WHO 2018; WHO 2019). The change was made after several studies found the new combination to induce a more rapid viral suppression, higher barrier to drug resistance, and lower potential for drug-drug interactions (Paul et al 2020; Kandel and Walmsley 2015; Osterholzer and Goldman 2014) and better patient experience (Twimukye et al 2021).

The higher proportion of pork samples with ARV residues seen in the Dar es Salaam region is rather concerning given that the region receives supplies from all over the country, and might indicate widespread use of ARVs in animal farming. This finding coupled with the level of residues seen in the samples from the Iringa and Mbeya regions implies that a good number of community members are slowly exposed to sub-therapeutic levels of ARVs which might contribute to the emergence and spread of HIV drug resistance and other health issues including toxicity, allergic reactions, and carcinogenicity, as previously reported (Odey et al., 2023).

Tanzania has several legislations that are intended to safeguard animal farming activities to ensure the welfare of animals and address public safety issues. These include; the Veterinary Act No.16 2003, Animal Welfare Act 2008, and the Meat Industry Act 2006, operating in conjunction with the National Public Health Act 2009. However, none of these acts focus on ARV residues in animal products, which our results indicate are present. We recommend the following measures to safeguard animal and human health: i) raise public awareness of the negative effects of using ARVs in animal farming; ii) establish well-coordinated surveillance and monitoring systems for the residues using relatively cheaper technologies; iii) revise legislation and acts governing animal farming and products, iv) ensure the veterinary council of Tanzania and other regulatory agencies oversees veterinary professional ethics and coordinates the formulation of appropriate guidelines and standards; v) provide educational advancement of veterinary professionals, paraprofessionals, and paraprofessional assistants on this issue and associated risks; and vi) deploy relatively inexpensive technologies that can be used at the community level to detect low concentrations of analytes of a more broadened spectrum, including second-line ARVs.

In summary, the results of this study shed light on the use of ARVs and the presence of ARV residues in animal farming in Tanzania highlighting the public health threats and the need for immediate action. The strength of our study is that samples were analyzed through LC-MS/MS, a method capable of detecting even trace amounts of drug residues present in analyzed samples.

However, we acknowledge some limitations of our study including i) we only screened for three first-line ARVs and did not include any second-line ARVs; ii) we could not relate the occurrence of ARV residues with farming practices since the samples were collected at the abattoir/slaughter slabs, and iv) we did not analyze samples from the environment associated with animal farming to assess any spillover of ARVs into the environment through animal manure.

## Conclusion

Our study has confirmed the use of ARVs in domestic pigs and broiler chickens in Tanzania, with potential exposure of residues to humans and the threat of environmental contamination, with potentially severe consequences. There is a need for well-coordinated cost-effective measures to monitor ARV residues in animal products. Larger surveys are encouraged to determine the magnitude of the problem and reasons for this practice to guide the development and institution of proper One Health interventions. Such surveys should include qualitative methods to determine why ARVs are used in animal production, the sources of the ARVs, and knowledge, attitudes, and practices of farmers regarding ARV use in farming. Revision of the training curriculum and educational advancement of veterinary professionals, paraprofessionals, and paraprofessional assistants will be essential to change current practices. Finally, the regulations guiding animal production activities should be revised.

## Figures and Tables

**Table 1:** ARV reference standards used for data validation

Matrix	ARV Reference Standards	Retention time (min.)	Recovery (%)	LOD	LOQ
Samples of animal origins	Lamivudine (mg/kg)	0.90	120.37	0.001	0.01
	Nevirapine (mg/kg)	7.90	106.26	0.005	0.05
	Efavirenz (mg/kg)	8.40	105.72	0.005	0.05
Water	Lamivudine (mg/kg)	0.90	91.79	0.001	0.01
	Nevirapine (mg/kg)	7.90	71.68	0.005	0.05
	Efavirenz (mg/kg)	8.40	100.86	0.005	0.05
Animal feeds	Lamivudine (mg/kg)	0.90	91.79	0.001	0.01
	Nevirapine (mg/kg)	7.90	112.22	0.005	0.05
	Efavirenz (mg/kg)	8.40	100.86	0.005	0.005

**Table 2:** The distribution of lamivudine residues by region (n = 196).

Region	Samples collected	No of positive (%)	No of negative (%)
Dar es Salaam	124	91 (73.4%)	33(26.6%)
Mbeya	32	15 (47%)	17 (53%)
Iringa	40	25 (62.5%)	15(37.5%)

**Table 3:** Distribution of samples with ARV lamivudine residues per district (n = 196)

District	Samples collected	No. of positive (%)	No. of negative (%)
Kinondoni	70	58 (83%)	12 (17%)
Ilala	34	15 (44%)	19 (56%)
Temeke	20	18 (90%)	2 (10%)
Mbeya CC	15	9 (60%)	6(40%)
Mbeya DC	11	2 (18%)	9(82%)
Mbalizi	6	4 (66.7%)	2 (33.3%)
Iringa MC	28	16 (57%)	12 (43%)
Mafinga	7	6 (85.7%)	1 (14.3%)
Kilolo	5	3 (60%)	2 (40%)

**Table 4:** Distribution of occurrence of ARV lamivudine residues per sample type (n = 196)

Sample type	Sample collected	No. of positive (%)	No. of negative (%)
Broiler chicken meat	30	30 (100%)	0
Broiler chicken blood	20	10 (50%)	10 (50%)
Domestic pig meat	39	36 (92.3%)	3 (7.7%)
Domestic pig blood	49	49 (77.6%)	11 (22.4%)
Broiler chicken feed	17	15 (88.2%)	2 (11.8%)
Domestic pig feed	4	3 (70%)	1 (25%)
Water samples	37	0 (0%)	37 (100%)

**Table 5:** Concentration of ARV Lamivudine (mg/kg) based on sample types

Sample type	Lowest conc. (mg/kg)	Highest conc. (mg/kg)	Average conc. (mg/kg)
Broiler chicken meat	0.22	1.69	0.97
Broiler chicken blood	0.09	0.92	0.16
Domestic pig meat	0.07	7.58	2.92
Domestic pig blood	0.06	7.58	1.94
Broiler chicken feed	0.01	0.43	0.21
Domestic pig feed	0.14	0.22	0.12

## Data Availability

The datasets used and/or analyzed during the current study are available from the corresponding author upon reasonable request.
